# Improving Nursing Quality Through On‐Site Supervision: A Process Management Intervention Guided by the Structure–Process–Outcome Model

**DOI:** 10.1155/jonm/5565207

**Published:** 2026-10-02

**Authors:** Min Li, Yueqin Li, Maoqing Fan, Liping Cui

**Affiliations:** ^1^ Department of Nursing Shanxi Bethune Hospital Shanxi Academy of Medical Sciences Third Hospital of Shanxi Medical University Tongji Shanxi Hospital Taiyuan 030032 China

**Keywords:** clinical competency, nursing management, on-site supervision, process evaluation, quality improvement, SPO model

## Abstract

**Aim(s):**

To develop and evaluate an Structure–Process–Outcome (SPO) model‐based on‐site supervision program for nursing process management, and to assess its impact on nursing compliance, competency, patient satisfaction, and adverse events.

**Background:**

The SPO model provides a comprehensive framework for evaluating healthcare quality, yet its translation into practical on‐site nursing supervision tools remains limited. Nursing quality assessments frequently emphasize retrospective outcome indicators while offering limited insight into real‐time procedural adherence.

**Method(s):**

A pre–post quasi‐experiment was conducted in a tertiary hospital (January 2024–December 2025), comparing outcomes between independent annual cohorts of nurses (2024 vs. 2025) within the same wards. Participants were not individually matched across the two phases. The intervention included a “1 + 1 + *X*” supervision team (Structure), formative evaluation with a “Ten Knowledges” checklist (Process), and a closed‐loop feedback mechanism. Data were collected from nurses (*n* = 194) and patients (*n* = 155) and analyzed using *t*‐tests and chi‐square tests.

**Results:**

Nursing compliance (aseptic technique: 91.86% vs. 83.80%), skill scores (93.65 ± 2.28 vs. 91.80 ± 3.76), and patient satisfaction (97.44 ± 3.93 vs. 95.86 ± 5.26) improved significantly (*p* < 0.05). The annual number of documented nursing‐related adverse events decreased from 559 in 2024 to 398 in 2025 (presented as raw counts without adjustment for total procedures).

**Conclusion(s):**

The SPO‐based program was associated with improvements in nursing quality and patient safety indicators, suggesting a replicable strategy for nurse managers.

**Implications for Nursing Management:**

This program offers nurse managers a structured, theory‐driven framework for on‐site supervision that can be adapted across similar clinical settings. By strengthening procedural compliance, clinical competency, and patient‐centered care, the approach provides a practical strategy for improving nursing quality and patient safety within existing resource constraints.

## 1. Introduction

The pursuit of high‐quality nursing care is a cornerstone of healthcare systems globally, serving as a critical determinant of patient safety and outcomes [1]. Effective nursing quality management necessitates robust evaluation systems that assess not only end results but also the care delivery process itself. Current quality assessment frameworks in clinical practice, however, often exhibit a retrospective and outcome‐oriented bias. Evaluations tend to emphasize compliance with outcome indicators (e.g., fall rates, pressure ulcer incidence), while offering limited insight into real‐time adherence to standards during complex nursing procedures [2]. This oversight is significant, as research indicates that a substantial portion of adverse nursing events result from deviations in operational protocols and lapses in process execution [3]. This highlights the critical need for a focus on pre‐execution and procedural elements in quality improvement initiatives [4]. The Structure–Process–Outcome (SPO) model, proposed by Donabedian, provides a timeless and holistic framework for evaluating healthcare quality [5]. It posits that effective structures (resources, organization) support proper processes (clinical activities), which ultimately result in desired outcomes (health status, satisfaction). While the SPO model has been extensively applied in macro‐level healthcare evaluations, such as the development of health‐promoting hospital evaluation systems, its practical translation into an operational tool for on‐site, formative nursing supervision remains limited. Most clinical supervision relies on intermittent, checklist‐based audits, without a coherent theoretical linkage between organizational support, real‐time performance, and sustained improvement [6]. Furthermore, nursing staff often express the need for more effective and structured clinical training [7], particularly due to existing gaps in their understanding and practical application of nursing quality management tools. A prior SPO‐informed quality initiative conducted at the same institution examined procedural pathways in combination with information management for skills training. That investigation, however, focused on a different intervention—namely, the construction of a training system—whereas the current study targets real‐time on‐site supervision of clinical nursing procedures. The two investigations employed separate participant cohorts, distinct intervention strategies, different data collection intervals, and nonoverlapping outcome measures; no participants or datasets were shared between them.

To bridge this gap, the present study developed and implemented an innovative, SPO model–based on‐site supervision program specifically designed for nursing process management. Rather than using the SPO model solely as an evaluative lens, this program operationalizes it into an integrated intervention consisting of: (S) a dedicated “1 + 1 + *X*” supervision team structure; (P) a formative evaluation process utilizing a validated procedure‐specific checklist; and (O) a closed‐loop feedback mechanism that links findings to targeted improvement actions. This approach aims to shift supervision from a passive, retrospective audit to an active, developmental, theory‐driven quality improvement activity. The program directly addresses the need to enhance the quality and structure of on‐site supervision in nursing practice [8] and builds on the specialized training system for intensive care nurses to optimize team composition.

It is hypothesized that the implementation of this structured supervision program would be associated with improvements in (a) nursing operational compliance and clinical competency [9], (b) patient satisfaction, and (c) the incidence of operation‐related adverse events.

The present investigation extends our prior SPO‐informed work in several substantive respects. Whereas the earlier study centered on training system development, the current inquiry applies the SPO framework to real‐time clinical supervision of nursing procedures. A novel “1 + 1 + *X*” supervision team configuration and a formative “Ten Knowledges” assessment checklist—neither of which featured in the prior work—were introduced as core intervention components. Moreover, the earlier investigation relied on retrospectively assembled control data, whereas the present study employs a prospective pre–post design within the same clinical units across two consecutive years. These refinements represent a meaningful progression from training system construction to direct oversight of clinical practice, thereby addressing an identified gap in process‐oriented nursing quality management.

## 2. Methods

### 2.1. Conceptual Framework Guiding the Intervention

The design and implementation of the on‐site supervision program were explicitly guided by Donabedian’s SPO model. As depicted in Figure 1, this foundational model was translated into a three‐component intervention: (S) the establishment of a dedicated “1 + 1 + *X*” supervision team to provide organizational support, based on the vertical management model for specialized nurse training; (P) the application of formative, real‐time evaluations through a checklist derived from the “Ten Knowledges” framework; and (O) a process‐level closed‐loop feedback mechanism that systematically channels evaluation findings into targeted improvement actions. Within this reconfigured framework, the closed‐loop feedback is conceptualized as an integral component of the PROCESS dimension—rather than as an outcome in itself—since it functions to reinforce and sustain proper clinical practices. The measured clinical and organizational variables—namely, nursing compliance scores, skill assessment scores, patient satisfaction ratings, and adverse event counts—constitute the true Outcome dimension, representing the distal results of the structured supervision process. It was hypothesized that this integrated intervention would initially lead to improvements in nurses’ operational compliance and clinical competency. These proximal improvements would, in turn, contribute to distal outcomes such as enhanced patient satisfaction and a reduction in operation‐related adverse events.

**FIGURE 1:**
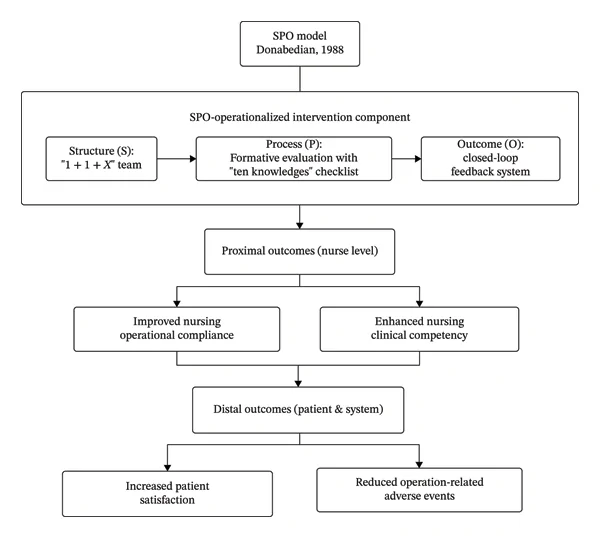
Conceptual framework of the SPO‐based on‐site supervision program. This figure illustrates the operationalization of the SPO model into intervention components and the hypothesized pathway to proximal and distal outcomes.

### 2.2. Study Design and Ethical Considerations

A quasi‐experimental study with a pre–post intervention design was conducted to assess the impact of the SPO‐based supervision program. This study received approval from the Institutional Ethics Committee of Shanxi Bethune Hospital (Approval No. YXLL‐2025‐112, granted on April 18, 2025) and was conducted in accordance with the Declaration of Helsinki. This approval encompassed all phases of the investigation, including baseline data collection (2024), intervention implementation (2025), and postintervention evaluation. The committee reviewed and approved the study protocol (Version V1.0, dated April 16, 2025) and granted a waiver of written informed consent for patients, given that the study presented minimal risk and involved routine clinical supervision procedures. Written informed consent was obtained from all participating nurses prior to their enrollment, while verbal consent was obtained from patients or their families. The study adhered to the guidelines set forth by the National Nursing Development Plan (2021–2025), which emphasizes quality improvement and clinical training [10].

### 2.3. Setting and Participants

The study was conducted in a large tertiary teaching hospital in China from January 2024 to December 2025. The intervention formally commenced on January 1, 2025. Baseline data for the 2024 period were collected prospectively as part of routine nursing quality monitoring activities; these data were not extracted retrospectively from existing records. All data collection procedures and audit instruments were standardized prior to the baseline phase and remained consistent across both study periods. Participants were recruited using a convenience sampling method. Convenience sampling was chosen for practical feasibility, as the intervention required the participation of all nurses in the selected wards to implement the unit‐level supervision program. The selected wards were representative of the hospital’s medical and surgical units in terms of nurse staffing levels and patient acuity.

#### 2.3.1. Nurses

Clinical nurses from selected medical and surgical wards who performed the supervised procedures were included. Nurses on extended leave during the study period were excluded. A total of 250 nurses were enrolled. Complete skill assessment data were available for 84 nurses in the 2024 cohort and 110 nurses in the 2025 cohort, totaling 194 independent observations; 56 nurses were excluded due to incomplete data. The exclusions were distributed across both study years and were primarily attributable to transfers to other departments, withdrawal from the training program, extended sick leave, and incomplete assessment records. The proportion of excluded nurses did not differ substantially between the 2 years, and the exclusions were unrelated to nurse performance or study outcomes. Missing data are, therefore, unlikely to have introduced substantial bias. These two cohorts comprised different individuals due to annual staff rotation, meaning that participants were not individually matched across the pre‐ and postintervention periods. Compliance checks included 437 observations (216 in 2024 and 221 in 2025), with multiple observations per nurse to capture routine practice patterns rather than one‐time performance. However, the presence of observers may have influenced nurse behavior (Hawthorne effect), which is acknowledged as a limitation. The sample characteristics are consistent with the demographic distribution of nursing staff in similar studies on training needs.

The 2024 and 2025 nurse cohorts were broadly comparable in terms of age, years of clinical experience, educational background, and departmental distribution. Patient characteristics were likewise similar between the 2 years, with no notable differences in age, sex, or primary diagnosis. Given this baseline comparability, no additional statistical adjustment was performed.

#### 2.3.2. Patients

Adult inpatients who received the nursing procedures under supervision were invited to participate in satisfaction surveys. Those with severe cognitive or communication impairments were excluded. Patient satisfaction surveys were also conducted with independent samples (66 in 2024 and 89 in 2025), with no patient participating in both surveys. For patients with chronic conditions (e.g., chronic heart failure), nursing interventions also referenced the principles of refined nursing to ensure consistency with clinical practice standards.

A formal sample size calculation was not performed prior to the study, as the sample was determined by the total number of nurses and patients available in the participating wards during the study period. This pragmatic approach is consistent with similar unit‐level quality improvement studies.

### 2.4. Intervention: The SPO‐Based On‐Site Supervision Program

The intervention was a multicomponent program explicitly structured according to the SPO framework, adapted for real‐time on‐site supervision.

#### 2.4.1. Structure (S)

Establishment of the “1 + 1 + *X*” supervision team: The team comprised the following members: (1) a nursing expert (master trainer), (2) a nursing department officer (process coordinator), and (*X*) clinical teaching supervisors from various departments. Their responsibilities included protocol development, on‐site evaluation, feedback delivery, and coaching, drawing upon the team configuration used in specialized nurse training systems. The team held a weekly 30‐min calibration meeting to review evaluation consistency. All members completed a 4‐h training workshop on the use of the checklist prior to the intervention.

#### 2.4.2. Process (P)

Formative evaluation and on‐site supervision: Supervision was carried out using a Clinical Nursing Procedure Quality Assessment Form. This 41‐item checklist was developed based on national standards, clinical guidelines, and expert consensus. Its design was informed by the “Ten Knowledges” framework, which provides a comprehensive structure for holistic patient assessment (see Figure 2), encompassing dimensions related to psychological nursing competency. Content validity was established through a panel of five experts, yielding a content validity index (CVI) of 0.92 at the item level and 0.94 at the scale level. Interrater reliability was assessed during the pilot phase through 10 paired observations conducted by two independent supervisors on identical procedures, yielding a percent agreement of 89% and a Cohen’s kappa coefficient of 0.83 (95% CI: 0.72–0.94), indicating substantial interrater agreement. Internal consistency was examined using Cronbach’s alpha, which produced a value of 0.91 for the total checklist, suggesting excellent internal consistency. The checklist was pilot‐tested on two nonstudy wards over a 2‐week period, and minor wording adjustments were made on the basis of pilot feedback. These psychometric properties support the checklist’s reliability and validity for use in clinical supervision contexts. The on‐site process involved patient assessment observation, key step evaluation, and immediate structured feedback, aligning with the “early clinical exposure, multiple clinical rotations, and repeated clinical training” teaching model [11]. On‐site supervision was conducted at least twice monthly on each participating ward, with each observation session lasting approximately 30–45 min. A total of 221 postintervention observations were completed.

**FIGURE 2:**
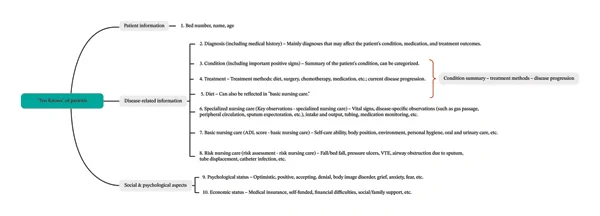
The “Ten Knowledges” framework for holistic nursing assessment (full name: ten core dimensions of comprehensive patient assessment). This figure depicts the comprehensive dimensions used to structure the checklist for formative evaluation during on‐site supervision.

#### 2.4.3. Outcome (O)

Measured clinical and organizational outcomes: The outcomes of this intervention were the clinical and organizational variables assessed before and after the intervention period, including nursing quality compliance scores, skill assessment scores, patient satisfaction, and adverse event counts. These variables were measured using standardized instruments and are reported in the Results section. The supervision process itself—encompassing documentation of findings, feedback delivery, and follow‐up audits—is conceptualized as part of the Process dimension, as it represents the ongoing mechanism through which practice standards are reinforced and sustained. A tiered feedback mechanism was implemented: issues were initially discussed with the individual nurse and head nurse, followed by departmental‐level analysis, and ultimately reported to the nursing administration for the development of systemic action plans. Follow‐up audits were conducted to verify corrections, completing a closed‐loop quality improvement cycle.

### 2.5. Measures and Variables

#### 2.5.1. Primary Outcome

Nursing quality compliance score: Eight core nursing quality dimensions were evaluated using hospital‐standardized audit tools developed by the hospital’s Nursing Quality Management Committee. Each audit tool comprises 8–12 items specific to the dimension under assessment (e.g., medication administration: 10 items; fall prevention: 8 items), with scores transformed to a 0–100 scale. The tools were adapted from the National Health Commission’s Nursing Quality Standards and underwent expert review by five senior nursing administrators, with content validity indices exceeding 0.85 across all dimensions. The tools have been employed in routine hospital‐wide audits since 2021, with established reliability and scoring criteria documented in the hospital’s quality management manual. Each dimension score ranged from 0 to 100. The count of nine in the original manuscript was a typographical error; the correct number is eight, as reflected in Table 1.

**TABLE 1:** Comparison of nursing quality scores between independent 2024 and 2025 cohorts.

Time period	*n*	Medication administration	Health education	Nursing documentation	Fall prevention	Pressure ulcer prevention	Catheter security	Blood transfusion	Pain management
2024	93	95.66 ± 3.17	96.16 ± 2.92	96.35 ± 3.03	95.56 ± 3.03	96.19 ± 3.03	96.65 ± 3.22	96.68 ± 2.83	96.45 ± 3.01
2025	93	96.95 ± 2.85	97.47 ± 2.52	97.19 ± 2.35	96.84 ± 2.64	97.18 ± 2.31	97.99 ± 2.24	97.70 ± 2.57	97.44 ± 2.50
*t*‐value	—	−2.919	−3.279	−2.110	−3.069	−2.501	−3.306	−2.579	−2.437
*p* value	—	0.004	0.001	0.036	0.002	0.013	0.001	0.011	0.016

*Note:* Nursing quality compliance scores were collected via hospital‐wide audits (*n* = 93 per year). Effect sizes (Cohen’s *d*) for significant *t*‐test comparisons ranged from 0.31 to 0.62 across the eight quality dimensions.

#### 2.5.2. Secondary Outcomes

Nursing skill assessment score: This was assessed using the aforementioned Clinical Nursing Procedure Quality Assessment Form, which provides total, procedural, and knowledge subscores. It includes items specifically measuring psychological nursing competency.

Operational compliance rates: Direct observation rates were assessed for four key practice standards: Operation Standard Compliance, Double‐check System Awareness, Aseptic Principle Implementation, and Hand Hygiene Compliance.

Patient satisfaction score: Measured using the Newcastle Nursing Satisfaction Scale (NSNS), an instrument originally developed by Thomas et al. (1996). The NSNS comprises 19 items rated on a 5‐point Likert scale (1 = *strongly disagree* to 5 = *strongly agree*), with total scores ranging from 19 to 95. The Chinese version has demonstrated sound psychometric properties, with a Cronbach’s alpha of 0.92 and test‐retest reliability of 0.87 in the validation sample. In the present study, the scale exhibited a Cronbach’s alpha of 0.90 at baseline and 0.91 at follow‐up. Subscale scores were computed for five domains: nursing time, nurses’ ability and competence, nurses’ visits to the ward, nurses’ affinity, and nurses’ respect for patients.

Adverse event count: the annual number of documented adverse events directly attributed to improper nursing operations was recorded.

### 2.6. Statistical Analysis

Data analysis was conducted using SPSS software (Version 26.0, IBM Corp.). Continuous variables from independent annual cohorts were compared using independent‐samples *t*‐tests, as the pre‐ and postintervention samples were not paired. Categorical variables were compared using the chi‐square test. Effect sizes for significant outcomes were calculated (Cohen’s *d* for *t*‐tests, Cramér’s *V* for *χ* ^2^ tests), and 95% confidence intervals were computed for all prespecified outcomes. To address the multiplicity arising from testing multiple outcomes, we applied a conservative Bonferroni correction, with a corrected significance threshold of *p* < 0.005 (0.05 divided by 10 outcomes). Outcomes with *p* values between 0.005 and 0.05 were interpreted as suggestive rather than definitive, which is consistent with statistical methods employed in similar nursing management studies [12]. A two‐tailed *p* value of less than 0.05 was considered statistically significant. Assessors were not blinded to the study phase, as the same supervision team conducted both baseline and follow‐up evaluations. To minimize bias, all assessors used a structured checklist with explicit criteria and underwent calibration training. For compliance observations involving multiple observations per nurse, the potential clustering effect was not adjusted for in the primary analysis due to the descriptive nature of these comparisons. This is acknowledged as a limitation.

## 3. Results

### 3.1. Comparison of Nursing Quality Compliance Scores

Table 1 presents an analysis of nursing quality scores before and after the intervention. In 2025, scores were significantly higher than in 2024 across all eight dimensions, with statistically significant differences observed (*t*‐values ranging from −3.306 to −2.110, *p* < 0.05).

### 3.2. Comparison of Nursing Operational Compliance Rates

Table 2 shows the differences in implementation rates before and after the intervention. In 2025, all nursing operational measures demonstrated higher compliance rates compared to 2024, with statistically significant differences (*p* < 0.05). This improvement coincided with the implementation of structured supervision, suggesting a potential association with enhanced application of nursing quality management tools.

**TABLE 2:** Comparison of nursing implementation rates between 2024 and 2025.

Time period	*n*	Operation standard compliance	Double‐check system awareness	Aseptic principle implementation	Hand hygiene compliance
2024	216	177 (81.94)	173 (80.09)	181 (83.80)	170 (78.70)
2025	221	200 (90.50)	194 (87.78)	203 (91.86)	192 (86.88)
*χ* ^2^ value	—	6.747	4.802	6.657	5.134
*p* value	—	0.009	0.028	0.010	0.023

*Note:* Effect sizes (Cramér’s *V*) for the four compliance indicators ranged from 0.10 to 0.12.

### 3.3. Comparison of Nursing Skill Assessment Scores

Table 3 presents the differences in assessment scores before and after the intervention. In 2025, overall assessment scores, procedural scores, related knowledge scores, and critical step scores were all significantly higher than in 2024 (*p* < 0.05). Notably, scores related to psychological nursing competency showed considerable improvement, suggesting a potential association with broader clinical competency development.

**TABLE 3:** Comparison of nursing assessment scores between 2024 and 2025.

Time period	*n*	Total assessment score	Operation score	Key procedure score	Related knowledge score	Patient sample (n)	Satisfaction score
2024	84	91.80 ± 3.76	94.01 ± 3.78	89.88 ± 5.98	87.06 ± 8.77	66	95.86 ± 5.26
2025	110	93.65 ± 2.28	95.03 ± 2.41	92.67 ± 5.85	90.48 ± 6.73	89	97.44 ± 3.93
*t*‐value	—	−3.989	−2.281	−3.256	−2.970	—	−2.061
*p* value	—	< 0.001	0.024	0.001	0.003	—	0.042

*Note:* (1) Nursing skill assessment data were collected from a subset of participating nurses (2024: *n* = 84; 2025: *n* = 110) due to resource constraints. (2) Patient satisfaction data were collected from adult inpatients who received supervised nursing procedures (2024: *n* = 66; 2025: *n* = 89), excluding those with severe cognitive or communication impairments. (3) The satisfaction survey included five items related to humanistic care (e.g., communication attitude, psychological support), with subscores increasing from 95.23 ± 4.89 (2024) to 97.12 ± 3.67 (2025) (*p* = 0.038). (4) Effect sizes (Cohen’s *d*) for significant comparisons were 0.62 for total assessment score, 0.32 for operation score, 0.51 for key procedure score, and 0.44 for related knowledge score.

### 3.4. Comparison of Adverse Events

The annual number of documented adverse events attributed to improper nursing operations declined from 559 in 2024 to 398 in 2025 (Figure 3). As these are raw counts without a denominator (e.g., total procedures or patient‐days), they are presented as descriptive counts rather than standardized incidence rates.

**FIGURE 3:**
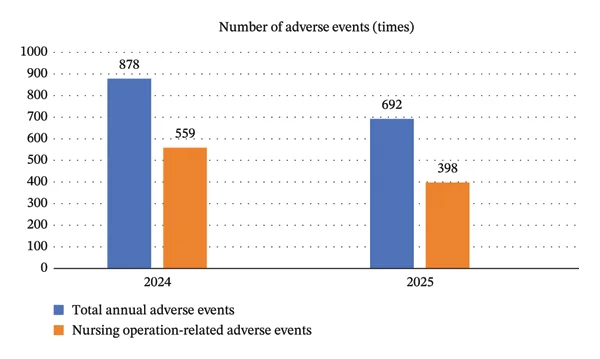
Annual counts of nursing operation‐related adverse events before and after the intervention. *Note:* This bar chart compares the documented annual counts of adverse events attributable to nursing procedures in the preintervention year (2024) and the postintervention year (2025). Data are presented as raw counts without adjustment for total procedures or patient volume.

## 4. Discussion

An SPO model–based on‐site supervision program for nursing process management was developed and rigorously evaluated in this study. The program directly addresses calls for improving the quality and structure of on‐site supervision in nursing practice [13] and responds to the identified need for more structured clinical training [14]. Our findings are consistent with the hypothesis, showing significant improvements in nursing quality compliance, operational standards, clinical competency, and patient satisfaction, as well as a reduction in adverse events. The reduction in raw counts should be interpreted cautiously, as we were unable to calculate incidence rates due to the unavailability of a reliable denominator across all procedure types. These findings underscore the value of applying a structured theoretical framework to on‐site supervision, aligning with the broader application of SPO theory in healthcare quality management [15].

First, procedural skill scores improved following the implementation of the structured supervision program. The “zero‐tolerance” items in the process (P) evaluation established clear safety boundaries, reinforcing critical standards. Retrospective reviews of recorded procedures provided opportunities for self‐reflection and learning, moving beyond fault‐finding to educational development [16]. The observed improvements in skill scores are consistent with the principle that timely, task‐specific feedback within a supportive environment can facilitate skill development [17]. The structured supervision process also appeared to support the application of learned skills in clinical practice [18].

Second, improvements were also observed in nurses’ procedural skills [19]. The structured supervision process, supported by a stable team framework, appeared to create conditions conducive to learning. This was reflected in the higher scores across all assessment components, including the knowledge subscore, which increased from 87.06 ± 8.77 to 90.48 ± 6.73 (Table 3). The process‐oriented nature of the supervision may have helped nurses integrate patient context and apply knowledge more flexibly in clinical situations [20]. The closed‐loop feedback system, with its follow‐up audits, provided a mechanism for consolidating learning and addressing recurring issues.

Third, the on‐site supervision process included patient interviews and assessment of psychosocial factors [21], alongside evaluation of technical procedures. This broader assessment scope was reflected in the humanistic care subscore of the patient satisfaction survey, which increased from 95.23 ± 4.89 to 97.12 ± 3.67 (*p* = 0.038) (Table 3, Note 3). The observed improvement in this subscore suggests that attention to communication and psychosocial dimensions in supervision may have contributed to more patient‐centered care delivery, consistent with the view that humanistic care is integral to nursing quality [22]. However, since psychological nursing competency was not measured as a stand‐alone scale, this finding should be interpreted with caution.

Fourth, the cognitive and application gaps in nursing quality management tools observed at baseline were less pronounced in the 2025 assessments. This is reflected in the improvement in nurses’ knowledge scores (Table 3: 87.06 ± 8.77 vs. 90.48 ± 6.73, *p* = 0.003), particularly in areas related to the application of these tools. By integrating these tools into the structured SPO supervision process, nurses gained hands‐on experience in utilizing management tools to enhance practice, rather than perceiving them as isolated audit instruments. This was accompanied by more frequent use of quality management tools in clinical practice, as noted by the supervising team.

These findings are consistent with previous studies on effective nursing training and quality improvement [23] and further support the application of the SPO model across diverse healthcare quality contexts [15].

### 4.1. Implications for Nursing Management

This study offers nurse managers a practical, theory‐driven blueprint for quality improvement, addressing the need for structured supervision and training [24]. The SPO‐based supervision program offers a structured framework that can be adapted to similar clinical settings, drawing on principles from specialized nurse training frameworks. The program shifts the focus from retrospective error identification to prospective process improvement. While implementing such a system entails initial investment in supervisor training and tool development, the observed improvements in compliance scores and patient satisfaction suggest potential benefits for nursing quality. However, claims regarding scalability and staff morale are not directly supported by the present data and warrant further investigation. Furthermore, the integration of psychological nursing competency assessments and attention to vulnerable patient groups ensures that the program aligns with contemporary nursing priorities.

### 4.2. Limitations and Future Research

This study has several limitations. The single‐center, pre–post design restricts generalizability and does not establish causality as definitively as a randomized controlled trial. Additionally, the use of convenience sampling may introduce selection bias. The presence of observers during compliance checks may have induced a Hawthorne effect, potentially overestimating routine performance. Additionally, the lack of assessor blinding may have introduced observer bias. These factors should be considered when interpreting the improvements. Baseline scores for several quality dimensions were already high (exceeding 95 out of 100 in 2024), which may have limited the room for substantial practical improvement despite achieving statistical significance. The clinical meaningfulness of these small‐magnitude changes requires further investigation. Future research should incorporate a randomized controlled trial design across multiple centers to better validate the intervention’s effectiveness. Long‐term follow‐up (e.g., 2 years) is necessary to evaluate the sustainability of improvements in nursing competency and adverse event rates. Adverse event data were collected as raw counts rather than incidence rates adjusted for exposure volume, which limits the ability to draw firm conclusions regarding safety improvement. Furthermore, exploring the model’s adaptability in specialized nursing fields (e.g., pediatric nursing, palliative care)—drawing on the experience of vertical management models for intensive care nurses [25]—could expand its applicability. Investigating the cost‐effectiveness of this supervision model would also provide valuable insights for managerial decision‐making. Notably, the long‐term impact of the intervention on vulnerable patient groups (e.g., those with chronic conditions) was not assessed and should be considered in future studies.

## 5. Conclusion

The SPO model–based on‐site nursing supervision program was associated with improvements in clinical nursing quality indicators in this single‐center study. By operationalizing Donabedian’s classic framework into a structured process of supervision, feedback, and system‐level improvement—while integrating insights from specialized nurse training [26], psychological nursing competency, and nursing quality management tools—the program was associated with improvements in addressing gaps in process‐oriented quality management. This approach not only improved immediate performance metrics but was also associated with a stronger emphasis on continuous learning and patient‐centered care during the study period. These findings suggest that the program may be worthy of broader application and adaptation in similar tertiary hospital settings, pending further validation through multicenter studies with extended follow‐up, aligning with national nursing development priorities [10]. The single‐center design and lack of a control group warrant caution in generalization.

## Author Contributions

Min Li: conceptualization, data curation, investigation, and writing–original draft. Yueqin Li: formal analysis, methodology, supervision, and writing–review and editing. Maoqing Fan: project administration and resources. Liping Cui: conceptualization, methodology, supervision, validation, and writing–review and editing.

## Funding

This work was supported by the 14th Five‐Year Plan Project (2025) of Shanxi Provincial Education Science (Topic) (Grant No.: GH‐25143).

## Disclosure

All authors read and approved the final manuscript.

## Ethics Statement

This study was approved by the Institutional Ethics Committee of Shanxi Bethune Hospital (Approval No. YXLL‐2025‐112) and conducted in accordance with the Declaration of Helsinki. Written informed consent was obtained from all participating nurses, and verbal consent was obtained from patients or their families. All patient data were anonymized (e.g., replacing names with unique codes) to protect privacy, in line with the Declaration of Helsinki.

## Consent

The authors have nothing to report.

## Conflicts of Interest

The authors declare no conflicts of interest.

## Supporting Information

Additional supporting information can be found online in the Supporting Information section.

## Supporting information


**Supporting Information** The following supporting information is provided: Supporting File 1: Ethics approval document (YXLL‐2025‐112). Supporting File 2: SQUIRE 2.0 reporting checklist.

## Data Availability

The datasets generated and analyzed during the current study are not publicly available due to hospital privacy policies but are available from the corresponding author (Liping Cui; Email: cuiliping@sxbqeh.com.cn) on reasonable request.
