# Supplementary material for: Improving Nursing Quality Through On‐Site Supervision: A Process Management Intervention Guided by the Structure–Process–Outcome Model
Source: J Nurs Manag. 2026 Oct 2;2026:5565207. doi: 10.1155/jonm/5565207 (PMC13633458; doi:10.1155/jonm/5565207)
Supplement: Supplementary file 1 [file JONM-2026-5565207-s001.zip › Reporting checklist.docx]

The SQUIRE reporting checklist

For checking that reports that describe systematic work to improve the quality, safety and value of healthcare, using a range of methods to establish the association between observed outcomes and interventions. can be understood and used by everyone

How to use this reporting checklist

This reporting checklist allows authors to demonstrate that their manuscripts adhere to the [SQUIRE reporting guideline](https:/resources.equator-network.org/reporting-guidelines/squire/index.html).

If you have not used a reporting guideline before, read about [how and why to use them](https:/resources.equator-network.org/about/reporting-guidelines.html) and check whether SQUIRE is the [most applicable reporting guideline](https:/resources.equator-network.org/reporting-guidelines/squire/index.html?#applicability) for your work.

Reporting guidelines are most useful when used early in research. When writing a manuscript or application, consider using the [full guidance](https:/resources.equator-network.org/reporting-guidelines/squire/index.html) where you’ll find explanations and examples for each item.

After writing, demonstrate adherence by completing this checklist:

1. Specify where each item is described (see Note 1).
2. Cite this checklist (See Note 2).
3. Include your completed checklist as a supplement when submitting to a journal so that future readers can use it to find information.

|  | Item Description | Location (or reason for not reporting) |
| --- | --- | --- |
| **Title and Abstract** |  |  |
| [1. Title](https:/resources.equator-network.org/reporting-guidelines/squire/items/title.html?utm_source=squire&utm_medium=checklist&utm_campaign=SQUIRE_2_0_2016_v1_1) | Indicate that the manuscript concerns an initiative to improve healthcare (broadly defined to include the quality, safety, effectiveness, patient-centredness, timeliness, cost, efficiency and equity of healthcare). | Title page; Title: "Improving Nursing Quality through On-Site Supervision: A Process Management Intervention Guided by the SPO Model". |
| [2. Abstract](https:/resources.equator-network.org/reporting-guidelines/squire/items/abstract.html?utm_source=squire&utm_medium=checklist&utm_campaign=SQUIRE_2_0_2016_v1_1) | 2a. Provide adequate information to aid in searching and indexing.  2b. Summarise all key information from various sections of the text using the abstract format of the intended publication or a structured summary such as: background, local problem, methods, interventions, results, conclusions. | Abstract (Background, Aims, Methods, Results, Implications for Nursing Management). |
| **Introduction** |  |  |
| [3 & 4. Problem description & Available Knowledge](https:/resources.equator-network.org/reporting-guidelines/squire/items/problem-description-available-knowledge.html?utm_source=squire&utm_medium=checklist&utm_campaign=SQUIRE_2_0_2016_v1_1) | 3. Nature and significance of the local problem. 4. Summary of what is currently known about the problem, including relevant previous studies. | Introduction, first paragraph: describes the retrospective, outcome-oriented bias of current nursing quality assessments and the lack of real-time procedural adherence monitoring.  Introduction, first and second paragraphs: cites literature on nursing quality assessment limitations [1–4], the SPO model [5], existing supervision gaps [6], and nursing training needs [7]. |
| [5. Rationale](https:/resources.equator-network.org/reporting-guidelines/squire/items/rationale.html?utm_source=squire&utm_medium=checklist&utm_campaign=SQUIRE_2_0_2016_v1_1) | Informal or formal frameworks, models, concepts, and/or theories used to explain the problem, any reasons or assumptions that were used to develop the intervention(s), and reasons why the intervention(s) was expected to work. | Introduction, second paragraph: describes the SPO model as the guiding framework, operationalised as "1+1+X" supervision team (Structure), formative "Ten Knowledges" checklist evaluation (Process), and closed-loop feedback mechanism (Outcome). |
| [6. Specific aims](https:/resources.equator-network.org/reporting-guidelines/squire/items/specific-aims.html?utm_source=squire&utm_medium=checklist&utm_campaign=SQUIRE_2_0_2016_v1_1) | Purpose of the project and of this report. | Introduction, second paragraph: "To bridge this gap, the present study developed and implemented an innovative, SPO model-based on-site supervision program specifically designed for nursing process management." Introduction, third paragraph: hypothesis statement. |
| **Methods** |  |  |
| [7. Context](https:/resources.equator-network.org/reporting-guidelines/squire/items/context.html?utm_source=squire&utm_medium=checklist&utm_campaign=SQUIRE_2_0_2016_v1_1) | Contextual elements considered important at the outset of introducing the intervention(s). | Methods, Section 2.2 and 2.3: describes the setting (large tertiary teaching hospital), convenience sampling, and selection of wards representative of medical and surgical units. |
| [8. Intervention(s)](https:/resources.equator-network.org/reporting-guidelines/squire/items/interventions.html?utm_source=squire&utm_medium=checklist&utm_campaign=SQUIRE_2_0_2016_v1_1) | A. Description of the intervention(s) in sufficient detail that others could reproduce it B. Specifics of the team involved in the work. | Methods, Section 2.4: detailed description of Structure (1+1+X supervision team composition, training, calibration), Process (41-item checklist, Ten Knowledges framework, supervision frequency and duration), and Outcome (closed-loop feedback mechanism). |
| [9. Study of the Intervention(s)](https:/resources.equator-network.org/reporting-guidelines/squire/items/study-of-the-interventions.html?utm_source=squire&utm_medium=checklist&utm_campaign=SQUIRE_2_0_2016_v1_1) | A. Approach chosen for assessing the impact of the intervention(s) B. Approach used to establish whether the observed outcomes were due to the intervention(s). | Methods, Section 2.2 and 2.3: pre-post quasi-experimental design; independent annual cohorts (2024 vs 2025); comparison of outcomes between pre- and post-intervention cohorts. |
| [10. Measures](https:/resources.equator-network.org/reporting-guidelines/squire/items/measures.html?utm_source=squire&utm_medium=checklist&utm_campaign=SQUIRE_2_0_2016_v1_1) | A. Measures chosen for studying processes and outcomes of the intervention(s), including rationale for choosing them, their operational definitions, and their validity and reliability B. Description of the approach to the ongoing assessment of contextual elements that contributed to t… | Methods 2.5 Measures and Variables: describes primary/secondary outcomes; psychometric properties of the 41‑item checklist, Newcastle Nursing Satisfaction Scale and hospital‑standardised audit tools.  N/A: No pre‑specified formal assessment of contextual moderators or cost‑efficiency was performed; cost‑effectiveness analysis is recommended for future research. |
| [11. Analysis](https:/resources.equator-network.org/reporting-guidelines/squire/items/analysis.html?utm_source=squire&utm_medium=checklist&utm_campaign=SQUIRE_2_0_2016_v1_1) | A. Qualitative and quantitative methods used to draw inferences from the data B. Methods for understanding variation within the data, including the effects of time as a variable. | Methods 2.6 Statistical Analysis: independent‑samples t‑tests, chi‑square tests, Bonferroni correction, effect‑size calculation; notes unadjusted clustering as a limitation.  N/A: Only two discrete annual cohorts were compared; repeated time‑series variation analyses were not conducted. |
| [12. Ethical considerations](https:/resources.equator-network.org/reporting-guidelines/squire/items/ethical-considerations.html?utm_source=squire&utm_medium=checklist&utm_campaign=SQUIRE_2_0_2016_v1_1) | Ethical aspects of implementing and studying the intervention(s) and how they were addressed, including, but not limited to, formal ethics review and potential conflict(s) of interest. | Methods, Section 2.2: ethics approval (YXLL-2025-112, granted April 18, 2025), informed consent, waiver of written consent for patients. Declarations section: competing interests statement, funding disclosure. |
| **Results** |  |  |
| [13 a & b. Evolution of the intervention and details of process measures](https:/resources.equator-network.org/reporting-guidelines/squire/items/intervention-evolution-process-measures.html?utm_source=squire&utm_medium=checklist&utm_campaign=SQUIRE_2_0_2016_v1_1) | 13a. Initial steps of the intervention(s) and their evolution over time (e.g., time-line diagram, flow chart, or table), including modifications made to the intervention during the project.  13b. Details of the process measures and outcome. | No modifications were made to the intervention protocol during implementation.  Results, Sections 3.1–3.4: presents nursing quality compliance scores (Table 1), operational compliance rates (Table 2), skill assessment scores (Table 3), and adverse event counts (Figure 3). |
| [13 c, d & e Contextual elements and unexpected consequences](https:/resources.equator-network.org/reporting-guidelines/squire/items/context-unexpected-consequences.html?utm_source=squire&utm_medium=checklist&utm_campaign=SQUIRE_2_0_2016_v1_1) | 13c. Contextual elements that interacted with the interventions  13d. Observed associations between outcomes, interventions and relevant contextual factors  13e. Unintended consequences such as benefits, harms, unexpected results, problems or failures associated with the intervention(s) | N/A: No prospective data collection for interaction between contextual factors and intervention outcomes.  Discussion section: interprets associations between the structured supervision programme and observed improvements in compliance, skills, and patient satisfaction.  N/A: Unintended consequences were not prospectively measured. |
| [13 f. Missing data](https:/resources.equator-network.org/reporting-guidelines/squire/items/missing-data.html?utm_source=squire&utm_medium=checklist&utm_campaign=SQUIRE_2_0_2016_v1_1) | 13f. Details about missing data. | Methods 2.3 Setting and Participants: describes reasons for 56 incomplete nurse records and risk of missing‑data bias. |
| **Discussion** |  |  |
| [14. Summary](https:/resources.equator-network.org/reporting-guidelines/squire/items/summary.html?utm_source=squire&utm_medium=checklist&utm_campaign=SQUIRE_2_0_2016_v1_1) | 14a. Key findings, including relevance to the rationale and specific aims  14b. Particular strengths of the project | Discussion, first paragraph: summarises key findings consistent with hypothesis. Discussion, Implications section: addresses strengths of the structured framework. |
| [15. Interpretation](https:/resources.equator-network.org/reporting-guidelines/squire/items/interpretation.html?utm_source=squire&utm_medium=checklist&utm_campaign=SQUIRE_2_0_2016_v1_1) | 15a. Nature of the association between the intervention(s) and the outcomes  15b. Comparison of results with findings from other publications  15c. Impact of the project on people and systems  15d. Reasons for any differences between observed and anticipated outcomes, including the influence of context  15e. Costs and strategic trade-offs, including opportunity costs | Discussion: interprets associations; compares with previous studies [23][24] and aligns with SPO theory [24]; Implications section addresses impact on nursing management. Limitations section notes high baseline scores and potential modest clinical significance. |
| [16. Limitations](https:/resources.equator-network.org/reporting-guidelines/squire/items/limitations.html?utm_source=squire&utm_medium=checklist&utm_campaign=SQUIRE_2_0_2016_v1_1) | 16a. Limits to the generalisability of the work.  16b. Factors that might have limited internal validity such as confounding, bias, or imprecision in the design, methods, measurement, or analysis.  16c. Efforts made to minimise and adjust for limitations. | Discussion, Limitations section: single-centre design (generalisability), convenience sampling (selection bias), Hawthorne effect (observer presence), lack of assessor blinding (observer bias), raw adverse event counts without denominators (safety interpretation), high baseline scores (limited improvement room). Efforts to minimise bias: standardised checklists, calibration training, explicit criteria. |
| [17. Conclusion](https:/resources.equator-network.org/reporting-guidelines/squire/items/conclusion.html?utm_source=squire&utm_medium=checklist&utm_campaign=SQUIRE_2_0_2016_v1_1) | 17a. Usefulness of the work  17b. Sustainability  17c. Potential for spread to other contexts  17d. Implications for practice and for further study in the field  17e. Suggested next steps | Conclusion: programme associated with improvements; suggests broader application in similar tertiary settings; pending multi-centre validation with extended follow-up; recommends caution in generalisation due to single-centre design; suggests future research directions (RCTs, cost-effectiveness studies). |
| **Other information** |  |  |
| [18. Funding](https:/resources.equator-network.org/reporting-guidelines/squire/items/funding.html?utm_source=squire&utm_medium=checklist&utm_campaign=SQUIRE_2_0_2016_v1_1) | Sources of funding that supported this work. Role, if any, of the funding organisation in the design, implementation, interpretation and reporting. | Declarations, Funding section: supported by the 14th Five-Year Plan Project (2025) of Shanxi Provincial Education Science [Grant No. GH-25143]. The funding organisation had no role in study design, data collection, analysis, interpretation, or manuscript preparation. |

## 1 How to specify where content is

Tell the reader where they can find information. E.g.,

- Results; paragraph 2
- Methods, Participants; paragraphs 1 & 2.
- Table 3
- Supplement B, para. 4

If you have chosen not to describe an item, explain why. You can do this in the checklist, or as a note below it.

You can describe items in the article body, or in tables, figures, or supplementary materials, and should prioritize items you feel are most important to your intended audience. The order of items in your manuscript does not need to match the order of items in this checklist. You can decide how best to structure your work.

## 2 How to cite

Describe how you used SQUIRE at the end of your Methods section, referencing the resources you used e.g.,

‘We used the SQUIRE reporting guideline(1) to draft this manuscript, and the SQUIRE reporting checklist(2) when editing, included in supplement A’

If you use a reporting checklist, remember to include it as a supplement when publishing so that readers can easily find information and see how you have interpreted the guidance.

1.	Ogrinc G, Davies L, Goodman D, Batalden P, Davidoff F, Stevens D. SQUIRE 2.0 (standards for QUality improvement reporting excellence): Revised publication guidelines from a detailed consensus process. BMJ Quality & Safety [Internet]. 2016 Dec;25(12):986–92. Available from: <https://qualitysafety.bmj.com/content/25/12/986>

2.	Ogrinc G, Davies L, Goodman D, Batalden P, Davidoff F, Stevens D. The SQUIRE reporting checklist. In: Harwood J, Albury C, Beyer J de, Schlüssel M, Collins G, editors. The EQUATOR network reporting guideline platform [Internet]. The UK EQUATOR Centre; 2025. Available from: [https:/resources.equator-network.org/reporting-guidelines/squire/squire-checklist.docx](https://https:/resources.equator-network.org/reporting-guidelines/squire/squire-checklist.docx)
