# Supplementary material for: Improving Nursing Quality Through On‐Site Supervision: A Process Management Intervention Guided by the Structure–Process–Outcome Model
Source: J Nurs Manag. 2026 Oct 2;2026:5565207. doi: 10.1155/jonm/5565207 (PMC13633458; doi:10.1155/jonm/5565207)
Supplement: Supplementary file 1 [file JONM-2026-5565207-s001.zip › Ethics approval document.pdf]

医学伦理审查批件

IRB Review Approval Notice

批件号<br>Approval notice number	YXLL-2025-112		
项目类别<br>Project type	临床研究		
项目名称<br>Project name	基于SPO模型的护理操作质量督导在临床护理过程管理中的应用		
项目来源<br>Project declarer			
研究单位<br>Research department	山西白求恩医院 山西医学科学院		
主要研究者<br>Main researchers	康复医学科 栗敏		
审查类别<br>Review type	初始审查	审查方式<br>Review way	简易程序
审查日期<br>Review Date	2025年4月18日	审查地点<br>Address	NA
审查委员<br>Censors	宋 彬 范雪梅		
审查文件<br>Files	1. 医学伦理审查申请表<br>2. 豁免知情同意的申请<br>3. 负责人及团队的研究经历<br>4. 临床研究方案（版本号V1.0 版本日期2025.04.16）<br>5. 主要研究者GCP/伦理培训证书<br>6. 病例报告表CRF表/数据报告表<br>7. 研究者及研究团队利益冲突声明		

扫描全能王
3亿人都在用的扫描App

审查意见<br>Censors opinion	经伦理委员会审查认为允许上述项目开展。
年度/定期跟踪审查频率<br>Annual/regular tracking review frequency	批件有效期至2026年4月18日，请于到期前30天提交研究进展报告，报告内容包括：<br>1、研究的进展；<br>2、受试者纳入例数，完成例数，退出例数等；<br>3、确认严重不良事件及时上报，妥善处理；<br>4、可能影响研究风险受益的任何事件或新信息。<br>以下情况和事件要求研究者及时向伦理委员会报告，重新审查：<br>A、对方案的任何修改，其可能影响受试者权利、安全、福利或影响研究的实施；<br>B、与研究实施和研究产品有关的、严重的和意外的不良事件，以及研究者、申办者和管理机构所采取的措施；<br>C、可能影响研究受益/风险比的任何事件或新信息。
联系人与联系电话<br>Linkman and phone number	刘师伟 0351-8379146
主任委员签字/盖章<br>Commissioner signature	刘师伟
伦理委员会<br>IRB	山西白求恩医院 山西医学科学院<br>医学伦理委员会（盖章）
日期<br>Date	2025年4月18日

扫描全能王
3亿人都在用的扫描App
